# Long-term plasticity determines the postsynaptic response to correlated afferents with multivesicular short-term synaptic depression

**DOI:** 10.3389/fncom.2014.00002

**Published:** 2014-01-30

**Authors:** Alex D. Bird, Magnus J. E. Richardson

**Affiliations:** ^1^Warwick Systems Biology Centre, University of WarwickCoventry, UK; ^2^Warwick Systems Biology Doctoral Training Centre, University of WarwickCoventry, UK; ^3^School of Life Sciences, University of WarwickCoventry, UK

**Keywords:** long-term plasticity, short-term plasticity, synaptic depression, correlations and synchrony, voltage fluctuations

## Abstract

Synchrony in a presynaptic population leads to correlations in vesicle occupancy at the active sites for neurotransmitter release. The number of independent release sites per presynaptic neuron, a synaptic parameter recently shown to be modified during long-term plasticity, will modulate these correlations and therefore have a significant effect on the firing rate of the postsynaptic neuron. To understand how correlations from synaptic dynamics and from presynaptic synchrony shape the postsynaptic response, we study a model of multiple release site short-term plasticity and derive exact results for the crosscorrelation function of vesicle occupancy and neurotransmitter release, as well as the postsynaptic voltage variance. Using approximate forms for the postsynaptic firing rate in the limits of low and high correlations, we demonstrate that short-term depression leads to a maximum response for an intermediate number of presynaptic release sites, and that this leads to a tuning-curve response peaked at an optimal presynaptic synchrony set by the number of neurotransmitter release sites per presynaptic neuron. These effects arise because, above a certain level of correlation, activity in the presynaptic population is overly strong resulting in wastage of the pool of releasable neurotransmitter. As the nervous system operates under constraints of efficient metabolism it is likely that this phenomenon provides an activity-dependent constraint on network architecture.

## 1. Introduction

Synapses play a key role in transmitting and processing information throughout the nervous system and long-term shifts in synaptic efficacy are believed to underpin learning and memory (Hebb, 2002; Markram et al., 2011). Synapses function through release of neurotransmitters that then bind to receptors on the postsynaptic cell and transiently alter the membrane conductance. Neurotransmitters in the presynaptic terminal are stored and transported in vesicles (Fox, 1988; Hu et al., 2008). A number of vesicles are positioned at active sites where they have a certain probability of being released when the presynaptic cell spikes. Empty release sites are restocked after a variable period, with an overall rate of a few Hz (Südhof, 2004). Both the number of contacts per presynaptic cell and the activity in the presynaptic network can generate correlations in the release of neurotransmitter at synapses onto a single neuron; we demonstrate that postsynaptic activity is governed by a balance between these two sources of correlation.

The usage of vesicles due to presynaptic firing and stochastic replenishment means that the number of vesicles available for release is a highly dynamic quantity that is dependent on the history of afferent activity. In the immature cortex, the relatively high release probability and limited availability of vesicles causes a progressive reduction in synaptic efficacy during a period of sustained neuronal activity (Reyes and Sakmann, 1999; Chen and Buonomano, 2012). This short-term reduction in synaptic strength is known as vesicle depletion depression: an unstocked active site cannot induce a postsynaptic response to any incident action potential (Abbot, 1997; Tsodyks and Markram, 1997; Zucker and Regehr, 2002). The phenomenon is believed to play a role in gain control (Abbot, 1997; Abbott and Regehr, 2004; Rothman et al., 2009), information transmission (Zador, 1998; Kilpatrick, 2012; Scott et al., 2012), and adaptation to sensory stimuli (Furukawa et al., 1982; Hallermann and Silver, 2012). The synaptic plasticity models introduced by Abbot (1997) and Tsodyks et al. (1998) capture short-term depression accurately; they match empirical data and allow a richness of network behavior (Tsodyks et al., 1998) to emerge beyond that predicted by static synapses. Such models consider the mean efficacy of the synapse, averaged across several presentations of the same presynaptic stimulus; the predicted postsynaptic response therefore varies continuously. Several recent studies have considered a quantal model of synaptic function incorporating short-term depression, with probabilistic vesicle release and replacement to reflect trial-to-trial variability (Fuhrmann et al., 2002; de la Rocha and Parga, 2005; Rosenbaum et al., 2012). The impact of stochastic vesicle dynamics is particularly marked when mean synaptic drive is insufficient to bring the postsynaptic neuron to threshold and spiking activity is governed by fluctuations in the system (Gerstein and Mandelbrot, 1964; Kuhn, 2004). To induce postsynaptic firing in such a system it is necessary for the variable synaptic drive to exhibit coincidences; this occurs most regularly when that drive is correlated.

Correlations in neurotransmitter release between different sites can arise from two sources: from multiple contacts onto a postsynaptic neuron from the same presynaptic cell and from synchronous activity across the presynaptic population. The number of sites between a pair of neurons is fixed over short timescales, unlike the number of vesicles ready to release from the sites, but can vary widely over longer periods (Loebel et al., 2013) following potentiation or depression. Connections between neurons potentiate and depress in the long term chiefly through changes in this synaptic parameter—the number of independent release sites can be seen as a fundamental unit of memory. Synchronous firing in the presynaptic population emerges from the connectivity of neuronal networks (Aertsen et al., 1989) and has relevance for encoding sensory information (von der Malsburg, 1981; deCharms and Merzenich, 1996; Averbeck et al., 2006), motor control (Baker et al., 2001; Capaday, 2013) and decision making (Cohen and Newsome, 2008; Cain and Shea-Brown, 2013). Recent work suggests that modulation of correlations can be more significant for neuronal coding than alterations in the presynaptic firing rate (Seriès et al., 2004; Mitchell et al., 2009; Cohen and Kohn, 2011). Population synchronization is a transient phenomenon relative to the structural changes underlying long-term plasticity.

A detailed stochastic model of neurotransmitter dynamics at the presynaptic terminal is required to analyze the effects of presynaptic synchrony, particularly when long-term plasticity varies the structure of synapses through altering the number of release sites. It can be noted that multiple contacts between cells and transient correlations within a presynaptic population are likely to introduce considerable redundancy in the usage of vesicles: correlated events may lead to EPSPs many times larger than that required to reach threshold. However, evidence points to the nervous system operating under constraints of efficient metabolism (Levy and Baxter, 2002; Taschenberger et al., 2002; Savtchenko et al., 2012) suggesting such wastage would not commonly arise *in vivo*. It is therefore of interest to examine the effect on the postsynaptic cell of the interaction of partially synchronized afferent drive with multiple contacts per presynaptic cell. To this end, we analyze a model of a postsynaptic cell receiving input from a population of release sites distributed between different numbers of presynaptic neurons and with different levels of synchrony.

Following the basic model definitions, we first derive exact forms for the crosscorrelations of vesicle occupancies and release at multiple contacts from the same and different presynaptic cells. These correlations were previously derived by Rosenbaum et al. (2012) using a diffusion and additive-noise approximation, and our results show that this earlier method gave exact results for these quantities. We then go on to calculate the exact voltage mean and variance and, through comparison with the typical EPSP amplitude, argue that synaptic noise can become significantly non-Gaussian. We then derive two approximate limiting forms for the firing rate for low and high correlations and demonstrate that the postsynaptic response is optimal at intermediate levels of afferent correlations. We finally show that this effect is robust for neurons in which there is some level of synaptic homeostasis or soft limit on the total number of release sites.

## 2. Methods

**Figure 1:**
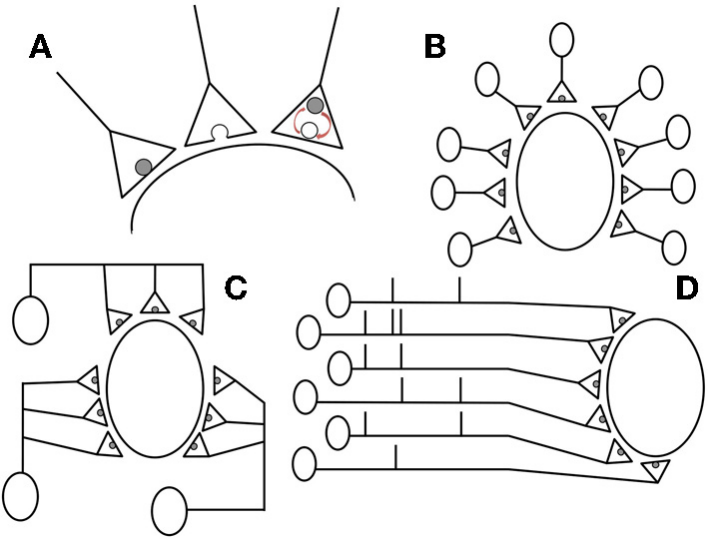
**We consider a population of *N* presynaptic neurons each featuring *n* independent release sites onto a single postsynaptic cell**. **(A)** The stochastic dynamics are illustrated from left to right: if a vesicle is present it is released (with probability *p*) when an action potential arrives (Poissonian rate *R*_*a*_); an empty release site; and restock of an empty release site (Poissonian rate *R*_*r*_). **(B,C)** examples with *M* = *nN* = 9 with **(B)** *n* = 1, *N* = 9 and **(C)** *n* = 3, *N* = 3 contacts and presynaptic neurons, respectively. **(D)** Example spike trains for *M* = *N* = 6 correlated presynaptic neurons that feature *S* = 3 synchronous spikes.

We consider a population of *N* presynaptic neurons synapsing onto a single postsynaptic neuron. A presynaptic neuron makes synapses with *n* vesicle occupancy sites from each of which neurotransmitter may be independently released with a probability *p* on the arrival of a presynaptic action potential, occurring at a constant Poissonian rate *R*_*a*_. In between presynaptic action potentials, empty release sites are restocked independently at a constant Poissonain rate *R*_*r*_. Initially, we consider that the total number of release sites onto the postsynaptic cell is fixed at *M* = *nN* (example configurations are provided in Figures 1A–C). The number of independent release sites *n* was recently shown (Loebel et al., 2013) to be the synaptic parameter most closely correlated with the structural changes arising from long-term plasticity and so we will consider the effects of varying *n* (while initially keeping *M* constant) on the postsynaptic response. The binary variable *x* will be used to signify vesicle release-site occupancy: *x* = 1 if present or *x* = 0 if absent. The evolution of vesicle occupancy is given by the stochastic differential equation

(1) $$\frac{dx}{dt}=(1-x)\sum_{m}\delta (t-t_{m})-\sum_{k}\varrho_{k}(x)\delta (t-t_{k})$$

where *m* counts the restock events occurring at a rate *R*_*r*_ and *k* counts the presynaptic action potentials occurring at a rate *R*_*a*_. The binary random variable ϱ_*k*_(*x*) signifies whether a release was successful at the *k*th action potential: if *x* = 1 then ϱ_*k*_(*x*) = 1 with probability *p* to model a successful release of neurotransmitter, and is 0 otherwise to model a failed release from a stocked site; if *x* = 0 then no release is possible and ϱ_*k*_(*x*) = 0. The δs are Dirac delta functions and whenever a delta function multiplies a dynamic variable it is assumed that the value of the variable used is that immediately before the delta event occurs. In other words, the equations are non-anticipating and should be interpreted in an Itō sense (Gardiner, 2010).

### 2.1. Correlations from structure

When a presynaptic neuron spikes, available vesicles at each of the *n* sites release their contents independently with probability *p*, and so the total number of release events is binomially distributed. Note that because these sites receive the same incoming action potentials correlations will arise despite the independent conditional release and restock events at each site. Globally, we first hold the total number of release sites, given by *M* = *nN*, constant so that the postsynaptic neuron receives a fixed overall excitatory drive. In this study we set *M* = 5000, which is of-the-order-of estimates by O'Kusky and Colonnier (1982), Megías et al. (2001), and Spruston (2008). This has the effect of maintaining the overall level of excitatory drive to the postsynaptic cell and in biological terms can be seen as a constraint of metabolic efficiency across the presynaptic population: as some contacts potentiate, others die out. The effects of relaxing this condition are discussed later. Recent analysis of long-term plasticity data has shown that changes in EPSP amplitude are captured by models in which the number of independent release sites *n* increases or decreases. Depending on the protocol, *n* can potentiate or depress by a factor of 5 or more (Loebel et al., 2013); a typical range for *n* is 5–50. However, contacts with a binomial *n* as low as 1 or as high as 100 sites have also been observed. Though the upper bound is unbiological, for completeness we vary *n* between 1 and 5000 in simulations.

### 2.2. Correlations from presynaptic synchrony

The population of neurons driving a common target often displays substantial synchrony in spiking activity (Salinas and Sejnowski, 2000; Averbeck et al., 2006; Cohen and Kohn, 2011) (see Figure 1D). Here we model correlations in the presynaptic population by using a variation of the Multiple Interaction Process (MIP) introduced in Kuhn et al. (2003). We implement the process by considering a master spike train with a constant Poissonian rate *NR*_*a*_/*S*. For each spike in the master train we pick *S* of the presynaptic neurons at random and assign a synchronous spike in their trains. If *S* = 1 this would imply no correlations in the presynaptic population and *S* = *N* would be a fully synchronous presynaptic population. Note that the spiking of each presynaptic neuron is Poissonian at rate *R*_*a*_ as required and also that, given that one presynaptic neuron spikes, the probability that a particular other presynaptic neuron has a spike at the same time is *c* = (*S* − 1)/(*N* − 1). In reality, shared spikes will not be entirely synchronous and so in later simulations (specifically, those leading to Figures 6, 7) we add independent, normally distributed jitter to the spike times with mean 0 and standard deviation τ_*j*_ following de la Rocha and Parga (2005) and Cohen and Kohn (2011). Note that in Figures 5, 6A,B, 7 the curves are truncated for increasing *n* because, for fixed *S* and fixed *M* = *nN*, it is invalid to have *S* greater than *N*. This is also the case for Figures 6B,C with increasing *S*.

### 2.3. Postsynaptic voltage

We treat the postsynaptic neuron as a leaky integrate-and-fire model with each neurotransmitter release event causing the voltage to jump by an amount *a*. The membrane voltage *V* has a resting value *E* and a spike threshold *V*_*th*_. After a spike, *V* is reset to *E* and held there for a time τ_*r*_ to model the refractory period. If *N* presynaptic neurons each have *n* neurotransmitter release sites then the postsynaptic voltage is governed by

(2) $$\tau \frac{dV}{dt}=E-V+a\tau \sum_{i=1}^{N}\sum_{j=1}^{n}\sum_{k}\varrho_{k}^{ij}(x_{ij})\delta (t-t_{k}^{i})$$

where τ is the membrane time constant, *x*_*ij*_ is the occupancy variable for the *i*th presynaptic neuron's release site number *j* and *k* labels the order of incoming action potentials to release site with occupancy *x*_*ij*_. Note that the spike times *t*^*i*^_*k*_ are identical for all release sites with the same presynaptic neuron *i* and that some of the spike times will be common to release sites with distinct presynaptic neurons, depending on the level of synchrony given by the correlated MIP process parameterized by *S*. The values of other parameters used in simulations (unless otherwise stated) are given in (Table 1).

**Table 1:** **Typical parameters used for the figures**.

**Parameter**	**Interpretation**	**Value**
*V*	Postsynaptic membrane voltage	Varies
*S*	Number of presynaptic cells that fire together	Varies
*n*	Number of release sites per presynaptic neuron	Varies
*N*	Number of presynaptic neurons	Varies
*M*	Total number of vesicle release sites (*nN*)	5000
*R*_*r*_	Rate at which empty vesicles are replaced at release sites	2Hz
*R*_*a*_	Rate of presynaptic spiking	2Hz
*p*	Probability of spike arrival inducing neurotransmitter release at a site with a vesicle present	0.66
τ_*j*_	Jitter standard deviation timescale	2ms
*E*	Resting membrane voltage	−70mV
*V*_*th*_	Threshold at which action potentials are initiated	−55mV
τ_*r*_	Refractory period of a neuron after a spike	2ms
τ	Membrane time constant	10ms
*a*	EPSP amplitude induced by neurotransmitter released from a single vesicle	0.2mV

## 3. Results

We first derive exact forms for the crosscorrelations of vesicle-occupancy and of neurotransmitter-release time series. The latter can then be used to calculate the exact membrane voltage variance. Two approximations of the postsynaptic firing rate then lead us to the main result of the paper: that long-term synaptic plasticity—through its alternation of the synaptic parameter *n*—sets the optimal postsynaptic response to a presynaptic population with correlated firing. Throughout this section the notation 〈ϕ〉 denotes the steady-state expectation of the fluctuating quantity ϕ.

For the calculation of the crosscorrelations of objects separated by a time *T*, it is useful to consider how the steady-state expectation of the product of the occupancy *x* with some quantity ψ evaluated at an earlier time evolves with the separation time:

(3) $$\frac{d}{dT}\langle x(T)\psi (0)\rangle =\langle (1-x(T))\psi (0)R_{r}\rangle -\langle x(T)\psi (0)\rangle pR_{a}$$

where the first term on the right-hand side is the rate that an empty site is filled and the second term is the rate that a full site releases its contents. This equation can be rearranged into the form

(4) $$\tau_{x}\frac{d}{dT}\langle x(T)\psi (0)\rangle =\langle x\rangle \langle \psi \rangle -\langle x(T)\psi (0)\rangle$$

where the time constant τ_*x*_ and steady-state occupancy 〈*x*〉 are

(5) $$\tau_{x}=\frac{1}{R_{r}+pR_{a}}\text{ and }\langle x\rangle =\frac{R_{r}}{R_{r}+pR_{a}}.$$

That the second quantity must be the steady-state occupancy 〈*x*〉 can be inferred by noting that in the limit *T* → ∞ the expectation 〈*x*(*T*)ψ(0)〉 in Equation (3) loses its *T* dependence and factorises into the product 〈*x*〉〈ψ〉. Note that the exponential solution to the differential Equation (4) implies that all crosscorrelations that include the occupancy *x* take a simple exponential form

(6) $$\text{Crosscorr}(x,\psi )=(\langle x\psi \rangle -\langle x\rangle \langle \psi \rangle )e^{-t/\tau_{x}}$$

where 〈*x*ψ〉 is the expectation evaluated in the limit *T* → 0.

### 3.1. Vesicle occupancy crosscorrelations

The autocorrelation of release-site occupancy can be calculated by making use of the fact that for the binary variable *x* we have *x*^2^ = *x* and so 〈*x*^2^〉 = 〈*x*〉. Putting ψ = *x* in equation (6) gives

(7) $$\text{Autocorr}(x)=\langle x\rangle (1-\langle x\rangle )e^{-|T|/\tau_{x}}=\frac{pR_{a}R_{r}}{{\left(R_{r}+pR_{a}\right)}^{2}}e^{-|T|/\tau_{x}}$$

where the extension of the exponential to negative times comes from a symmetry argument. For the crosscorrelation between different release sites, with occupancy variables *x* and *x*′, we need to distinguish between cases where the release sites either share the same presynaptic neuron or have different presynaptic neurons when deriving 〈*xx*′〉. However, the derivation can be written in the same form by introducing a quantity γ that is the proportion of shared spikes: γ = 1 for release sites with the same presynaptic neuron or γ = *c* = (*S* − 1)/(*N* − 1) for different presynaptic neurons. A steady-state equation for the zero-time expectation 〈*xx*′〉 can be found by considering the state where both sites are occupied and balancing the total rates into and out of this state

(8) $$\langle x(1-x^{\prime })\rangle R_{r}+\langle (1-x)x^{\prime }\rangle R_{r}=\langle xx^{\prime }\rangle (2R_{a}p-\gamma R_{a}p^{2}).$$

The terms on the left-hand side represent the total rate into the double occupancy state, whereas the terms on the right-hand side multiplying the expectation are the combined rates of individual vesicle release minus the coincidence term to prevent overcounting of events. We now combine terms to obtain the required expectation

(9) $${\langle xx^{\prime }\rangle }_{\gamma }=\frac{2R_{r}\langle x\rangle }{2R_{r}+R_{a}p(2-\gamma p)}$$

where the γ subscript will be used later to distinguish the different cases. It can be inserted into Equation (6) with ψ = *x*′ to give

(10) $$\text{Crosscorr}(x,x^{\prime })=\frac{\gamma p^{2}R_{a}R_{r}^{2}e^{-|T|/\tau_{x}}}{(2R_{r}+pR_{a}(2-p\gamma )){\left(R_{r}+pR_{a}\right)}^{2}}.$$

**Figure 2:**
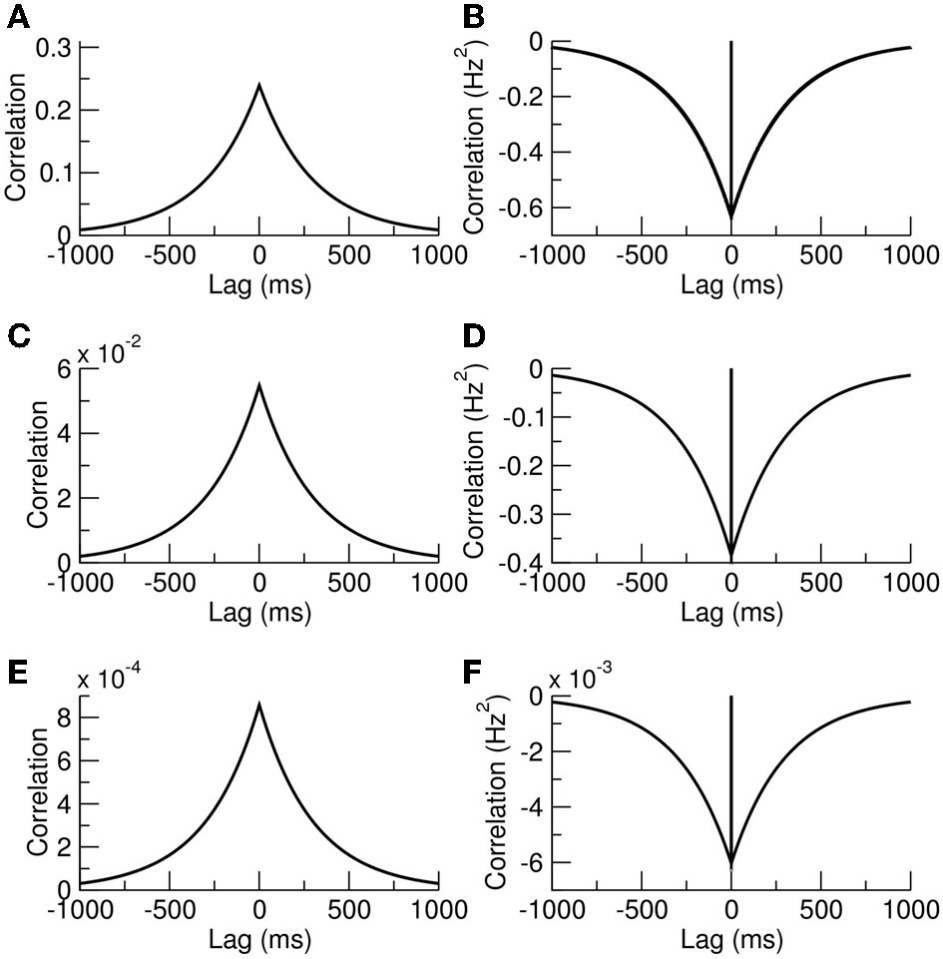
**Release-site occupancy is correlated, neurotransmitter-release events are anticorrelated**. **(A)** Autocorrelation of a release-site occupancy and **(B)** autocorrelation in neurotransmitter release. **(C,D)** Crosscorrelations for distinct release sites sharing the same presynaptic cell. **(E,F)** Crosscorrelations for release sites with different presynaptic cells. The parameters were *N* = 500, *n* = 10, and *S* = 10 giving the probability of synchronous spikes *c* = 0.018.

Example plots of Equation (7), and Equation (10) for cases with γ = 1 and γ = *c* are given in Figures 2A,C,E. It is interesting to note that our exact results are identical to those previously calculated in Rosenbaum et al. (2012) using a combined diffusion and additive-noise approximation, validating their method up to second-order statistics.

### 3.2. Neurotransmitter release crosscorrelations

Though synchrony in the presynaptic population leads to positive correlations for release-site occupancy, we now show that the delayed restock following release leads to negative cross-correlations in the release events themselves. Let χ(*t*) and χ′(*t*) be trains of delta pulses representing neurotransmitter release from sites with occupancies defined by *x*(*t*) and *x*′(*t*), respectively, so that:

(11) $$\chi (t)=\sum_{k}\varrho_{k}(x)\delta (t-t_{k})$$

where *k* counts incoming action potentials at the contact with site occupancy *x*. In the steady state we have 〈χ〉 = *pR*_*a*_〈*x*〉 because the rate of release is equal to the release rate *pR*_*a*_ given vesicle occupancy multiplied by the occupancy probability 〈*x*〉. The auto and crosscorrelations can be straightforwardly calculated using the general result of Equation (6) by setting ψ = χ′ and noting that 〈χ(*T*)χ′(0)〉 = *pR*_*a*_〈*x*(*T*)χ′(0)〉. However, some care needs to be taken when considering the case *T* = 0. The result of Equation (6) is valid in the limit *T* → 0; but there is an additional delta function in the crosscorrelation when *T* = 0 with an amplitude equal to the rate of simultaneous events in χ and χ′ that arises from the delta functions in Equation (11). The autocorrelation function for χ therefore takes the form

(12) $$\text{Autocorr}(\chi )=pR_{a}\langle x\rangle \delta (T)-{\left(pR_{a}\langle x\rangle \right)}^{2}e^{-|T|/\tau_{x}}$$

where the rate of simultaneous events for the autocorrelation is just the mean release rate *pR*_*a*_〈*x*〉 and prefactor of the exponential is only −〈χ〉^2^ because in the limit *T* → 0 the expectation of 〈χ(*T*)χ(0)〉 is zero as there is no time for a restock. A similar consideration gives the result for the crosscorrelation

(13) $$\begin{array}{l} \text{Crosscorr}(\chi ,\chi^{\prime })=\gamma p^{2}R_{a}{\langle xx^{\prime }\rangle }_{\gamma }\delta (T)\\ \;+R_{a}^{2}p^{2}((1-\gamma p){\langle xx^{\prime }\rangle }_{\gamma }-{\langle x\rangle }^{2})e^{-|T|/\tau_{x}} \end{array}$$

where we are treating cases for which the release is from distinct contacts sharing the same presynaptic neuron γ = 1 or from distinct presynaptic neurons where γ = *c*. In Equation (13) the prefactor of the delta function arises from the rate of simultaneous releases, which is equal to the arrival of simultaneous spikes γ*R*_*a*_ multiplied by the probability that each contact releases a vesicle *p*^2^〈*xx*′〉_γ_. The prefactor of the exponential shares the same squared component −〈χ〉^2^ = −(*pR*_*a*_〈*x*〉)^2^ as the autocorrelation, but also has a non-zero contribution from 〈χ(*T*)χ′(0)〉 in the limit *T* → 0. This quantity is equal to the probability that both sites are occupied 〈*xx*′〉_γ_ multiplied by the probability of a release from site *x*′ but no release from site *x* from a simultaneous presynaptic event, which is *R*_*a*_*p*(1 − γ*p*) multiplied by a subsequent release from site *x* just afterwards due to a second presynaptic spike, *pR*_*a*_. This exact result is again identical to that derived previously using a diffusion and additive-noise approximation (Rosenbaum et al., 2012). Example autocorrelation and crosscorrelation functions are plotted in Figures 2B,D,F.

### 3.3. Membrane voltage mean and variance

The tonic component of the presynaptic drive can be characterized by the mean voltage, which is straightforward to calculate in the absence of a threshold. The dynamics of this quantity can be found by taking the expectation of Equation (2) to yield the steady-state result

(14) $$\langle V\rangle =E+aM\tau pR_{a}\langle x\rangle =E+\frac{aM\tau pR_{a}R_{r}}{R_{r}+pR_{a}}.$$

Note that the mean voltage is independent of the synchrony *S* and is also independent of release-site number *n* when *M* = *nN* is held fixed.

The effect of correlated synaptic fluctuations on the postsynaptic neuron can also be characterized by deriving the steady-state variance of the postsynaptic voltage (again in the absence of a threshold-reset mechanism). This quantity is derived in the Appendix using the auto and crosscorrelations of χ (Equations 12, 13) and takes the form

(15) $$\begin{array}{l} \text{Var}(V)=\frac{a^{2}\tau NnpR_{a}}{2}(\langle x\rangle +(n-1)p{\langle xx^{\prime }\rangle }_{1}+(N-1)ncp{\langle xx^{\prime }\rangle }_{c})\\ \;+\frac{Nn{\left(a\tau pR_{a}\right)}^{2}}{1+\tau R_{r}+p\tau R_{a}}((n-1)(1-p){\langle xx^{\prime }\rangle }_{1}.\\ \;+(N-1)n(1-cp){\langle xx^{\prime }\rangle }_{c}-Nn{\langle x\rangle }^{2}). \end{array}$$

**Figure 3:**
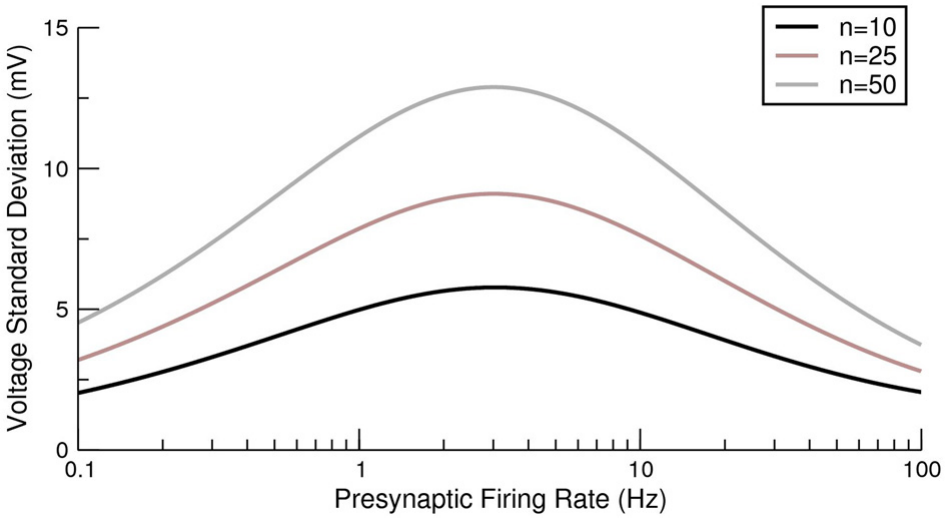
**Exact voltage variance for a postsynaptic neuron receiving multiple depressing synaptic contacts from a presynaptic population**. Three examples are given with different numbers of neurostransmitter release sites per presynaptic neuron. For each case the synchrony was *S* = 10.

The first term arises from the δ-functions in Equations (12, 13) and the second term comes from the negative correlations in vesicle release due to short-term depression (the terms featuring exponentials in the same equations). For a related model (de la Rocha and Parga, 2005) it was demonstrated that on increasing the presynaptic rate a maximum can be seen in the conductance fluctuations. The exact result of Equation (15) allows for this effect of fluctuations in depressing synapses on the voltage itself to be analyzed. Example variances as a function of presynaptic rate are shown in Figure 3 and, as expected from the previous analysis of conductance fluctuations (de la Rocha and Parga, 2005), the variance also shows a maximum at intermediate presynaptic rates.

Though the voltage variance measures one aspect of presynaptic fluctuations, it misses its increasing shot-noise nature as the correlations increase. Shot noise causes a non-Gaussian component in the tails of the membrane voltage distribution that, because they extend to the region of action-potential initiation, can significantly affect the post-synaptic firing rate (Richardson and Swarbrick, 2010). The mean EPSP amplitude can be used to see this effect: it is proportional to the mean of the vesicles released by a spike given the occupancy levels already computed, and so

(16) $$\langle \text{EPSP}\rangle =apnS\langle x\rangle =\frac{apSnR_{r}}{R_{r}+pR_{a}}.$$

**Figure 4:**
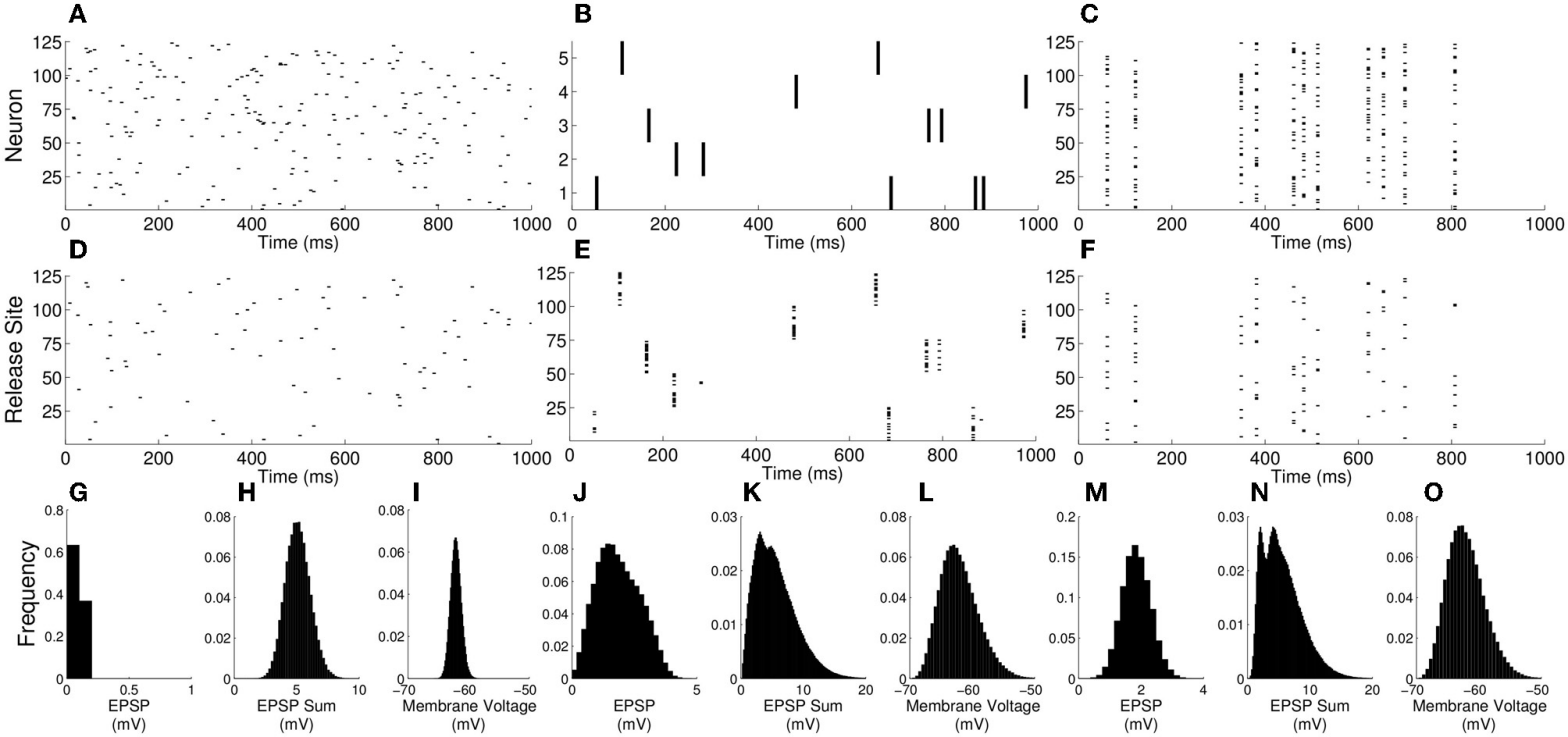
**Membrane voltage distributions become markedly non-Gaussian as correlations increase**. **(A–C)** Rasters of presynaptic firing with: **(A)** *N* = 125, *n* = 1, and *S* = 1; **(B)** *N* = 5, *n* = 25, and *S* = 1; **(C)** *N* = 125, *n* = 1, and *S* = 25. **(D–F)** Rasters of neurotransmitter release for these firing patterns. **(G,J,M)** EPSP histograms for the above *n* and *S* values, but with *N* adjusted so that *M* = *nN* = 5000. **(H,K,N)** Histograms of the total synaptic drive over an interval of the membrane time constant for the same parameters. **(I,L,O)** Voltage histograms for the same parameters. Note that, whereas the voltage is close to Gaussian for the single release-site and no-presynaptic-synchrony case, it develops a tail to the right when correlations arise either from multiple release sites or presynaptic synchrony.

As correlations from increasing *n* or *S* become stronger, the mean EPSP amplitude increases. However, as noted above, the mean voltage (Equation 14) does not change under increasing *n* or *S*. Taken together, the implications are that in the limit of high correlations the synaptic drive becomes temporally sparse with large amplitude EPSPs generated from correlated events. This effect can be seen in simulations of the model with different parameter regimes (Figure 4). For parameters *N* = 125, *n* = 1, and *S* = 1 (no presynaptic synchrony) the presynaptic spikes (Figure 4A) and neurotransmitter release (Figure 4D) are uncorrelated, and in the full system with *M* = 5000 the EPSPs are relatively small (Figures 4G,H) and the resulting voltage distribution is close to Gaussian (Figure 4I). Increasing *n* (Figure 4B) or *S* (Figure 4C) to 25 leads to correlations in neurotransmitter release (Figures 4E,F), larger EPSPs (Figures 4J,K,M,N) and a more variable and skewed membrane voltage (Figures 4L,O). Note the right-hand tails from the skewed membrane voltages under conditions of presynaptic correlation that extend toward voltages where action potentials would be initiated.

### 3.4. Release site number and postsynaptic rate

As the analyses of the previous section and examples in Figure 4 demonstrate, for the case of few release sites and low synchrony the voltage distribution is close to Gaussian. However, for the case of many release sites the synchronous release events generate large EPSPs that are reminiscent of shot noise. With this in mind, approximations for the firing of the postsynaptic cell may be found for the cases of low *n*, when the voltage distribution is roughly Gaussian, and high *n* for which the EPSP amplitudes are of-the-order-of or larger than threshold.

#### 3.4.1. Few release sites

For the low *n* approximation we rely on a recent observation (Alijani and Richardson, 2011) that the firing rate of integrate-and-fire neurons is relatively insensitive to temporal correlations as long as the subthreshold voltage mean and variance are matched. To this end we approximate the firing rate of the neuron by a white-noise equivalent that has a voltage mean μ equal to that of Equation (14) and variance σ^2^ equal to that of Equation (15). The firing rate of a leaky-integrate-and-fire neuron with these parameters is given (Brunel and Hakim, 1999) by the reciprocal of

(17) $$\tau \int_{0}^{\infty }\frac{dz}{z}e^{-z^{2}/2}(e^{zz_{th}}-e^{zz_{re}})$$

where *z*_*th*_ = (*V*_*th*_ − *E* − μ)/σ and in this case *z*_*re*_ = −μ/σ.

#### 3.4.2. Many release sites

For sufficiently large *n* the mean EPSPs are greater than that required to bring the neuron to threshold *apnS* 〈*x*〉 » *V*_*th*_ − *E*, and so each synchronous presynaptic event is likely to cause the postsynaptic cell to spike. The postsynaptic cell receives input at a total rate of *NR*_*a*_/*S* and so we can approximate the rate in the large *n* case by

(18) $$r\sim \frac{NR_{a}}{S}=\frac{MR_{a}}{nS}.$$

Therefore, increasing the presynaptic synchrony *S* will reduce the postsynaptic response when *n* is large.

#### 3.4.3. Optimal release-site number

**Figure 5:**
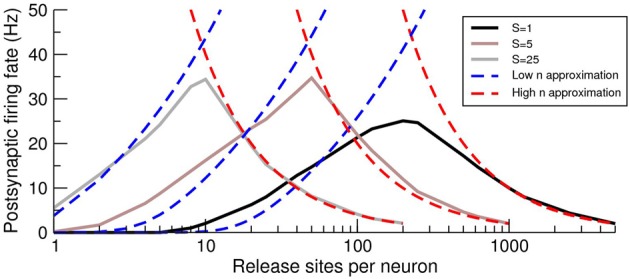
**The postsynaptic firing rate exhibits a maximum as a function of the number of pre-to-post release sites *n***. Firing-rate simulations (solid lines), low *n* approximation (Equation 17; blue dashed lines) and high *n* approximation (Equation 18; red-dashed lines) for various levels of presynaptic synchrony *S* as a function of the number of release sites *n* per presynaptic cell. The maximal postsynaptic response is close to the intersection of the approximate forms and the optimum *n* decreases with increasing synchrony *S*. Note that the curves are limited on their right because of the restriction *S* ≤ *N* (the maximal allowable synchrony is equal to the number of presynaptic neurons) so that the maximum *n* is *n* = *M/S*. This upper bound on *n* holds for similar curves in later figures.

Under conditions of a fixed number of release sites onto the postsynaptic cell *M* = *nN*, increasing *n* has no effect on the voltage mean (Equation 14), but increases the voltage variance (Equation 15). Therefore, as *n* increases from an initially small value, the approximation given by Equation (17) predicts that the postsynaptic cell will fire at an increasing rate. However, from Equation (18), which is valid for high *n*, we see that the postsynaptic firing rate decreases as *n* increases. Hence, there must be an intermediate *n* for which the response of the postsynaptic cell is optimized. This effect can be clearly seen in the examples given in Figure 5 in which the postsynaptic rate is plotted as a function of *n* for fixed *M*. The intersections of the two approximations for each curve provide an estimate for the optimal *n*, which decreases as the presynaptic synchrony increases. It should be noted that this effect, which has a maximum as a function of release-site number at constant presynaptic rate, is a distinct phenomenon to the tuning curve as a function of presynaptic rate analyzed in de la Rocha and Parga (2005).

### 3.5. Long-term plasticity and response to synchrony

**Figure 6:**
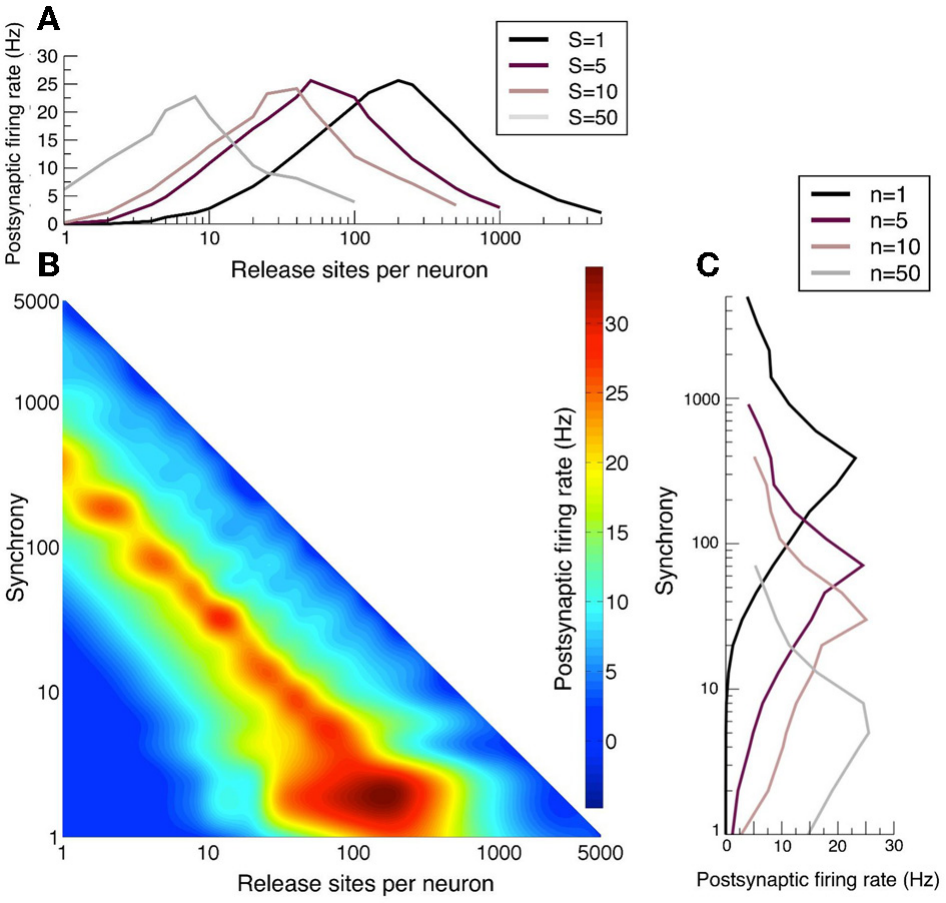
**Long-term plasticity that alters release-site number *n* sets the sensitivity to presynaptic synchrony**. **(A)** Postsynatpic rate as a function of release-sites per presynaptic neuron *n* for different examples of presynaptic synchrony *S*. **(B)** Heat map of the postsynaptic rate as a function of presynaptic release-site number *n* and presynaptic synchrony *S*. **(C)** Postsynaptic rate as a function of presynaptic synchrony *S* for different examples of release-site number *n*. For these figures population spikes have been jittered with a standard deviation of 2 ms. Note that in panel **B** the optimal synchrony has an inverse dependency on the release-site number. Long-term potentiation makes the postsynaptic cell more sensitive to weak synchrony, whereas long-term depression sensitizes the cell to stronger synchrony.

The post-synaptic firing rate is sensitive to correlations arising from multiple release sites, as discussed above, as well as to presynaptic synchrony (de la Rocha and Parga, 2005). In particular, the firing rate has a maximal response at an optimal *n* that is a function of the presynaptic synchrony as can be seen in Figure 6. When neurotransmitter release is too strongly correlated in the presynaptic population, the postsynaptic response weakens because the quantity of neurotransmitter released is in excess of that necessary to take the postsynaptic cell to threshold and therefore this limited resource is wasted. The reduction in response to over-strong correlations gives rise to the optimal responses in the space of *n* and *S* seen in Figures 6A–C. Note that the band of optimal postsynaptic response is linear with negative gradient in the *n, S* log–log plot and so the optimal synchrony in the presynaptic population has an inverse relation to the number of release sites *n* each presynaptic cell makes onto the postsynaptic target.

Analyses of long-term plasticity data (over a 12 h period) by Loebel et al. (2013) demonstrated that connections between thick-tufted layer-5 pyramidal cells in the rat somatosensory cortex alter their efficacy by changing the binomial parameter *n*, in preference to probability of release or quantal amplitude. Among the experiments analyzed certain connections potentiated four-fold, from an effective binomial *n* of ~25 to ~100. Assuming that the mean excitatory drive remains constant, this potentiation would lead to the postsynaptic cell becoming maximally responsive to signals encoded by weaker presynaptic synchrony (see Figure 6C). It would also cease to amplify strongly correlated stimuli as effectively. Other connections showed four-fold reductions in *n* from ~40 to ~10 under protocols that cause long-term depression. In this case the postsynaptic cell would now act as a better amplifier of stimuli encoded with larger correlations.

### 3.6. Optimal response and synchrony jitter

**Figure 7:**
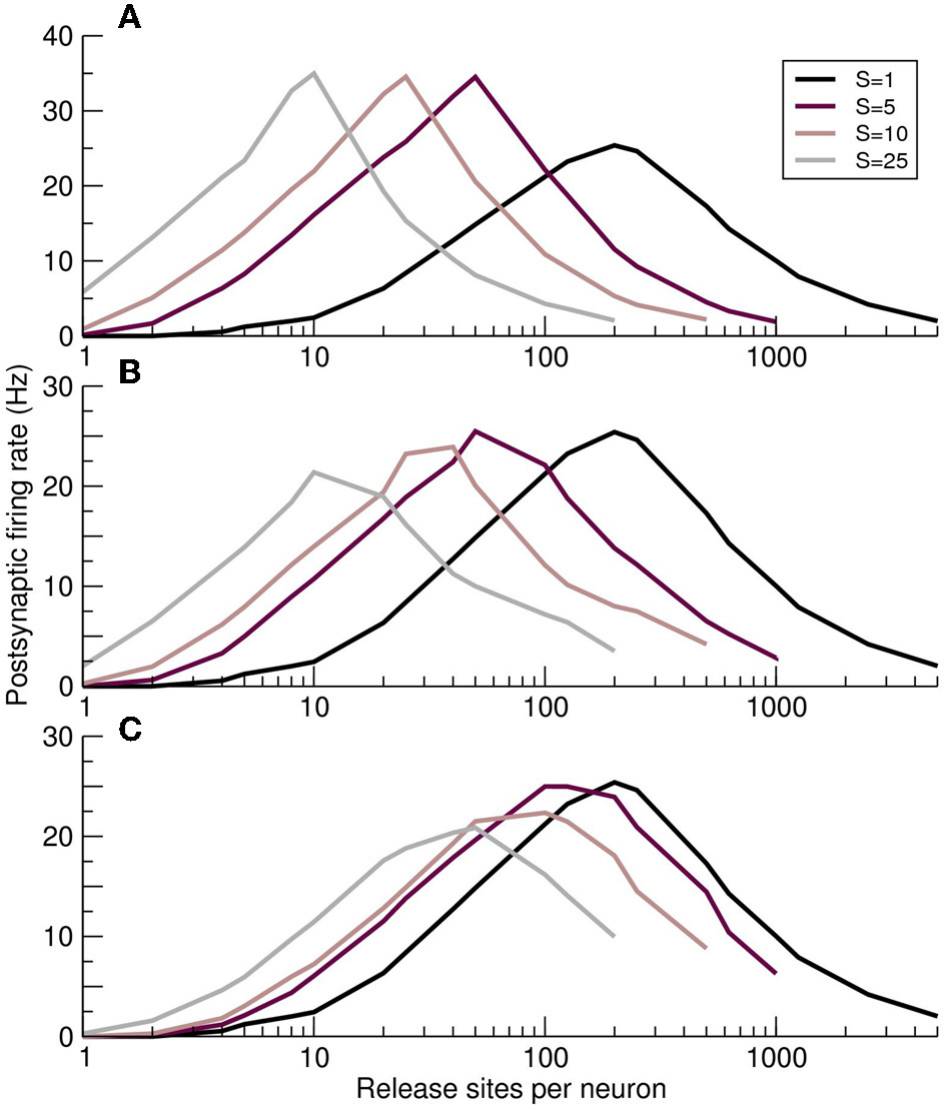
**Impact of synchrony jitter on the optimal response curves**. **(A–C)** Postsynaptic firing rate as a function of the number of release sites per presynaptic neuron *n* for increasing jitter standard deviations τ_*j*_. **(A)** No jitter τ_*j*_ = 0. **(B)** Physiological levels of jitter τ_*j*_ = 2ms. **(C)** Response curves converge on the unsynchronized *S* = 1 case, as expected, when jitter is of the order of the postsynaptic membrane time constant τ_*j*_ = 10ms.

The effects of fluctuations in a synchronous presynaptic population can be modeled by adding a Gaussian-distributed jitter, of timescale τ_*j*_, to the timing of each action potential. When the individual components of the synchronous MIP event are too dispersed temporally, i.e., when the jitter is greater than the membrane time constant τ_*j*_ > τ, the MIP event will fail to integrate in the postsynaptic cell. Under these circumstances the effect of correlations is diminished, as illustrated in Figure 7. When jitter is absent (Figure 7A), different values of presynaptic synchrony *S* produce distinct and clearly defined optimal response curves. With a physiological jitter timescale of τ_*j*_ = 2 ms (Figure 7B) the curves for different synchronies shift upwards in *n* and the peak postsynaptic firing rate falls, particularly for larger synchrony. When τ_*j*_ = τ (Figure 7C) only relatively strong synchrony values are significantly different from the independent case (*S* = 1).

### 3.7. Optimal-response curves are a robust feature of synaptic homeostasis

**Figure 8:**
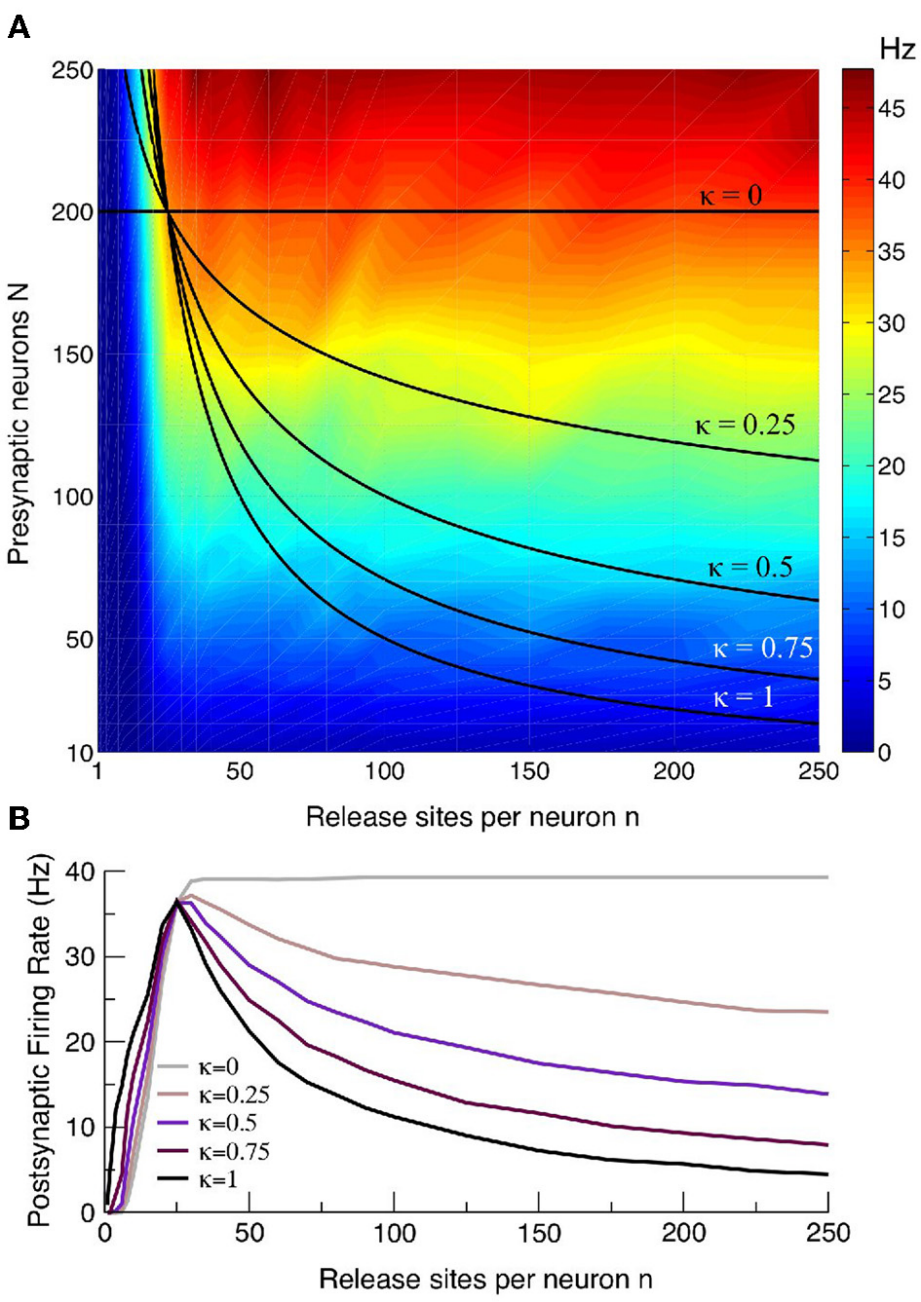
**Curves with a maximal postsynaptic rate at intermediate *n* are a robust feature**. **(A)** Intensity plot of the postsynaptic rate as a function of presynaptic release site *n* and neuron number *N* for an example with *S* = 10. Also plotted are the relations *N* = *M*_κ_/*n*^κ^ for κ = 0, 1/4, 1/2, 3/4, 1, where κ = 1 corresponds to the homeostatic scenario principally considered in this paper for which there is a restriction *M* = *nN* on the total number of afferent contacts. The case κ = 0 corresponds to a scenario with no such restriction, and the other values of κ are intermediate cases with varying degrees of homeostasis. **(B)** The postsynaptic rate as function of *n* for the curves in the upper panel. Cases for all values of κ, except κ = 0 in which there is no homeostatic restriction, show a maximal response at intermediate *n*. The example curves given have *M*_κ_ chosen so that they all pass through the point *n* = 25 and *N* = 200.

Throughout much of the above analysis we held the total number of release sites *M* = *nN* constant and demonstrated an optimal response curve in which the postsynaptic rate peaks at an intermediate *n*, which is dependent on the presynaptic synchrony *S*. The rationale for this choice is that, under conditions of homeostasis, synaptic potentiation (increasing *n*) amongst a subpopulation of presynaptic neurons will occur at the expense of pruning neurons that do not contribute to postsynaptic firing. This will lead to the postsynaptic neuron receiving afferent drive from fewer presynaptic neurons, though each of these will make more contacts (and vice-versa for long-term depression). The theoretical results and simulations are not predicated on the assumption of constant *M* and so it is interesting to investigate whether the optimal-response effect persists if this restriction is relaxed. Using the example *S* = 10 we plotted the postsynaptic rate as a function of the presynaptic neuron *N* and release site number *n* (see Figure 8A). As expected the postsynaptic rate increases with an increasing number of presynaptic neurons *N* or release sites *n*. Plotted on the same figure is the curve *N* = *M/n* with *M* = 5000 that, because of its reciprocal relation will have low rates at either asymptotes, and an intermediate maximum (see Figure 8B). Also plotted is the curve *N* = *M*_0_ where *M*_0_ is a constant. This corresponds to a scenario in which the entire presynaptic population has either potentiated or depressed their contacts, thereby changing the number of release sites *n* a presynaptic neuron makes without altering the total number of presynaptic neurons *N*. For this case, which is arguably an extremum from the point-of-view of homeostatis, the intermediate maximum is lost: the postsynaptic rate increases monotonically and loses its *n* dependence when *n* is sufficiently large, as expected from the first form of Equation (18). However, for intermediate cases of homeostasis of the form *N* = *M*_κ_/*n*^κ^ with κ = 3/4, 1/2, 1/4 a maximal postsynaptic rate again occurs at some intermediate *n* (see Figure 8B). Given the dependence of the postsynaptic rate on *n* and *N* in Figure 8A it can be seen geometrically that any curve in which there is a reciprocal relation between *N* and *n* will likely feature a maximum at intermediate *n* and so the optimal-response curves are a robust feature of a postsynaptic neuron in which there is some degree of homeostatic restriction on the total number of afferent contacts.

## 4. Discussion

We considered the effects of afferent correlations arising from multiple neurotransmitter release sites and a partially synchronized presynaptic population. We derived exact forms for the crosscorrelations of vesicle release site occupancy and vesicle release, and demonstrated that these are identical to those recently obtained from a diffusion and additive-noise approximation (Rosenbaum et al., 2012), validating that approach up to second-order statistics and explaining their perfect agreement between theoretical and simulational results. We further calculated the exact variance of the membrane voltage, in absence of spike threshold. This quantity extends previous calculations (de la Rocha and Parga, 2005) of synaptic conductance fluctuations and allows for an estimation of the postsynaptic rate in the low-correlation Gaussian regime. For the high-correlation regime, due to multiple release sites *n* or strong synchrony *S*, we argued that the EPSPs become increasingly large, the nature of the synaptic fluctuations increasingly shot-noise like, and so the postsynaptic rate tends to the rate of synchronous presynaptic events. Combing these two results for the low and high correlation regimes, we demonstrated that the postsynaptic response is maximal for an intermediate number of release sites or synchrony. The system therefore exhibits a tuning-curve response to synchrony that can be modulated by long-term plasticity, which alters the number of release sites *n*.

Neurons respond maximally to specific stimuli when processing sensory input. A coordination of long-term plasticity, afferent synchrony and short-term depression therefore provides a potential tuning mechanism for cells to achieve this sensitivity. Efficient responsiveness would then depend on historical changes in synaptic connectivity (Taschenberger et al., 2002; Loebel et al., 2013) and the transient correlations evoked by a particular stimulus (Averbeck et al., 2006; Cohen and Kohn, 2011). More generally, neuronal networks balance fidelity of signal transmission with the metabolic costs associated with neurotransmitter recycling (Levy and Baxter, 2002; Savtchenko et al., 2012). Although a release of neurotransmitter beyond that necessary to induce a postsynaptic spike may have medium-term conductance implications or counteract strongly fluctuating inhibition, an efficient network would not be expected to exceed the degree of pairwise connectivity that maximizes response to common spike frequencies and correlations. On the other hand, signals encoded by small numbers of cells would require highly potentiated connections to transmit information with any degree of consistency. This implies that across a neuronal network the degree of clustering would be optimally balanced with individual synaptic weights.

To investigate maximal firing rate response to a defined excitatory drive, we have neglected the effects of synaptic inhibition. As *in vivo* network behaviors arise from a balance of excitation and inhibition, a development of the ideas presented here along the above lines would need to incorporate inhibitory effects on the total synaptic conductance. By altering the timescales on which excitatory inputs are integrated, inhibitory drive could allow a more finely-tuned response to afferent sub-populations with varying degrees of temporal dispersion. Another extension of this work would be to incorporate different forms of short-term synaptic plasticity into the model. This would be particularly appropriate when studying connections between specific cell-types where there is experimental evidence for other forms of synaptic dynamics. It is also likely that effects moderating synaptic depression, such as the increasing facilitation in the maturing neocortex (Reyes and Sakmann, 1999) would lead to qualitatively different behavior as cortical networks develop.

## Funding

This research was supported by a Warwick Systems Biology Doctoral Training Centre fellowship to Alex D. Bird funded by the UK EPSRC and BBSRC funding agencies.

### Conflict of interest statement

The authors declare that the research was conducted in the absence of any commercial or financial relationships that could be construed as a potential conflict of interest.

## Appendix

### Derivation of the voltage variance

The voltage equation can be written in the form

(19) $$\tau \frac{dV}{dt}=E-V+a\tau \zeta$$

where ζ is the summation of the release trains across the *N* presynaptic neurons and each of their *n* contacts

(20) $$\zeta =\sum_{i=1}^{N}\sum_{j=1}^{n}\chi_{ij}$$

where χ_*ij*_ takes the form of Equation (11) for the *i*th presynaptic neuron's *j*th contact. The autocorrelation of ζ is therefore comprised of *Nn* autocorrelations of χ in the form of Equation (12), *Nn*(*n* − 1) crosscorrelations of χ for distinct release trains sharing the same presynaptic neuron given by Equation (13) with γ = 1 and *N*(*N* − 1)*n*^2^ crosscorrelations of χ for release trains with different presynaptic neurons given by Equation (13) with γ = *c*.

Taking expectations of both side of Equation (19) in the steady state gives

(21) $$\langle V\rangle =E+a\tau \langle \zeta \rangle =E+aM\tau R_{a}p\langle x\rangle .$$

We can now solve Equation (19) to give

(22) $$V-\langle V\rangle =a\int_{-\infty }^{t}dt^{\prime }e^{-(t-t^{\prime })/\tau }(\zeta (t^{\prime })-\langle \zeta \rangle )$$

so that the voltage variance can be written as an integral over the autocorrelation of ζ, Autocorr(ζ) = 〈(ζ(*t*′) − 〈ζ〉) (ζ(*t*″) − 〈ζ〉)〉

(23) $${\left(V-\langle V\rangle \right)}^{2}=a^{2}\int_{-\infty }^{t}dt^{\prime }\int_{-\infty }^{t}dt^{\prime \prime }e^{-(t-t^{\prime })/\tau }e^{-(t-t^{\prime \prime })/\tau }\text{Autocorr}(\zeta ).$$

As discussed above, the autocorrelation of ζ is the sum of the various crosscorrelations of χ so that it must take the form

(24) $$\text{Autocorr}(\zeta )=\alpha \delta (t^{\prime }-t^{\prime \prime })+\beta e^{-|t^{\prime }-t^{\prime \prime }|/\tau_{x}}$$

where α and β are obtained from the prefactors of the terms in Equations (12, 13) multiplied by their respective contributions. Inserting Equation (24) into (23) and performing the integration gives

(25) $$\text{Var}(V)=a^{2}(\frac{\alpha \tau }{2}+\frac{\beta \tau^{2}\tau_{x}}{\tau +\tau_{x}}).$$

On substituting the appropriate forms for α and β the result given in Equation (15) is obtained.
